# Acute and post-dosing effects of single-dose psilocybin for obsessive-compulsive disorder in a randomized, double-blind, placebo-controlled trial: an interpretative phenomenological analysis

**DOI:** 10.3389/fpsyt.2025.1726818

**Published:** 2025-12-10

**Authors:** T. H. W. Ching, B. Stahnke, S. Shnayder, G. Agin-Liebes, T. G. Adams, L. Amoroso, O. Baiz, A. Belser, C. Bohner, M. Burke, E. D’Amico, G. DePalmer, J. Eilbott, G. Fram, R. Grazioplene, J. Hokanson, A. Jankovsky, S. A. Kichuk, B. Martins, P. Purohit, H. Schaer, Y. P. Sierra, C. Witherow, C. Pittenger, B. Kelmendi

**Affiliations:** 1 Department of Psychiatry, Yale University School of Medicine, New Haven, CT, United States; 2 Department of Social Work, Eastern Tennessee State University, Johnson City, TN, United States; 3 New York University Postdoctoral Program in Psychoanalysis and Psychotherapy, New York, NY, United States; 4 Department of Psychology, Yale University, New Haven, CT, United States; 5 Center for Brain and Mind Health, Yale University School of Medicine, New Haven, CT, United States; 6 Child Study Center, Yale University School of Medicine, New Haven, CT, United States; 7 Department of Neuroscience, Yale University School of Medicine, New Haven, CT, United States; 8 Wu-Tsai Institute, Yale University, New Haven, CT, United States

**Keywords:** psilocybin, psychedelic, obsessive-compulsive disorder, acute effects, mental health, adult psychiatry, qualitative research, interpretative phenomenological analysis

## Abstract

**Introduction:**

The subjective effects of psilocybin on obsessive-compulsive disorder (OCD) are under-explored. Therefore, we conducted a qualitative study of participant experiences from the first randomized placebo-controlled trial of single-dose psilocybin combined with unstructured and non-directive support for individuals with treatment-refractory OCD. Our research explored how participants experienced acute and post-dosing effects, the interrelationships between these effects, and participants’ perspectives on therapeutic change.

**Materials and methods:**

We conducted qualitative interviews with 12 participants approximately one month after psilocybin dosing; (six who received psilocybin in the initial randomized placebo-controlled phase, six who received open-label psilocybin following unblinding). We analyzed interview transcripts via interpretative phenomenological analysis (IPA) and engaged in consensus decision-making to arrive at 100% intercoder agreement in the process of abstracting codes into higher-order themes.

**Results:**

Four major themes (and several subthemes) emerged from our analysis: 1) Influences on Psilocybin Experience (i.e., Set, Setting); 2) Acute Effects (i.e., Acute perceptual effects, Acute [meta]cognitive effects, Acute emotional effects, Acute impact of OCD, Other acute effects); 3) Post-Dosing Changes in OCD (i.e., Post-dosing changes in symptoms, Post-dosing changes in perceptions of OCD); as well as 4) Post-Dosing Changes Beyond OCD Symptoms (i.e., Post-dosing [meta]cognitive changes, Other post-dosing changes). Meaningful interrelationships among codes, subthemes, and themes were the norm.

**Discussion:**

Our findings highlight the moderate to strong influences of set and setting in the nature and trajectory of participants’ psilocybin experiences. We also uncovered acute, synergistic visual/perceptual, emotional/psychological, and physiological/somatic effects that map onto those commonly reported in prior psilocybin trials for other closely related indications. However, these acute effects tended to occur at lower intensities (i.e., ‘partial’ experiences) potentially due to acute interference by OCD symptoms. Certain acute and post-dosing (meta)cognitive and behavioral effects also map onto putative mechanisms of action in evidence-based psychotherapy for OCD (e.g., exposure and response prevention [ERP] and acceptance and commitment therapy [ACT]). These findings yielded hypotheses for future investigation, and point toward potential integration of psilocybin with structured psychotherapy approaches for OCD.

## 1 Introduction

Obsessive-compulsive disorder (OCD) is a prevalent mental disorder (1) characterized by recurrent, intrusive, and distressing obsessions, which often prompt repetitive, ritualized physical and mental compulsions in an effort to relieve or neutralize obsessional distress. OCD is chronically disabling (2–4), stigma-inducing (5), and associated with high latencies to treatment (6). While standard-of-care treatments for OCD such as selective serotonin reuptake inhibitors (SSRIs) and cognitive-behavior therapy with exposure and response prevention (CBT/ERP) are helpful for some (7–11), they continue to be limited by low remission (12–16) and high relapse rates (17, 18), highlighting the need for novel therapeutic approaches.

The resulting search for novel treatment alternatives dovetails with the revival of public, academic, and clinical interest in psychedelic treatments for refractory conditions. Psilocybin (4-phosphryloxy-N,N-dimethyltryptamine) is a serotonergic classic psychedelic (19–25) that has been examined in combination with psychological support as a promising treatment option in clinical trials for various disorders (26–28). Notably, the first post-prohibition psychedelic clinical trial completed in the US was a proof-of-concept, pilot study of repeated psilocybin dosing paired with unstructured psychological support for nine treatment-refractory participants with OCD (29). Results indicated that the protocol was safe, tolerable, feasible, with minimal to no serious adverse events (SAEs), and a short-term efficacy profile of 23 to 100% reduction in OCD symptoms 24 h after dosing. A recent retrospective online survey also found OCD symptom improvement to be uniquely associated with personal serotonergic psychedelic use (30).

**Figure 1:**
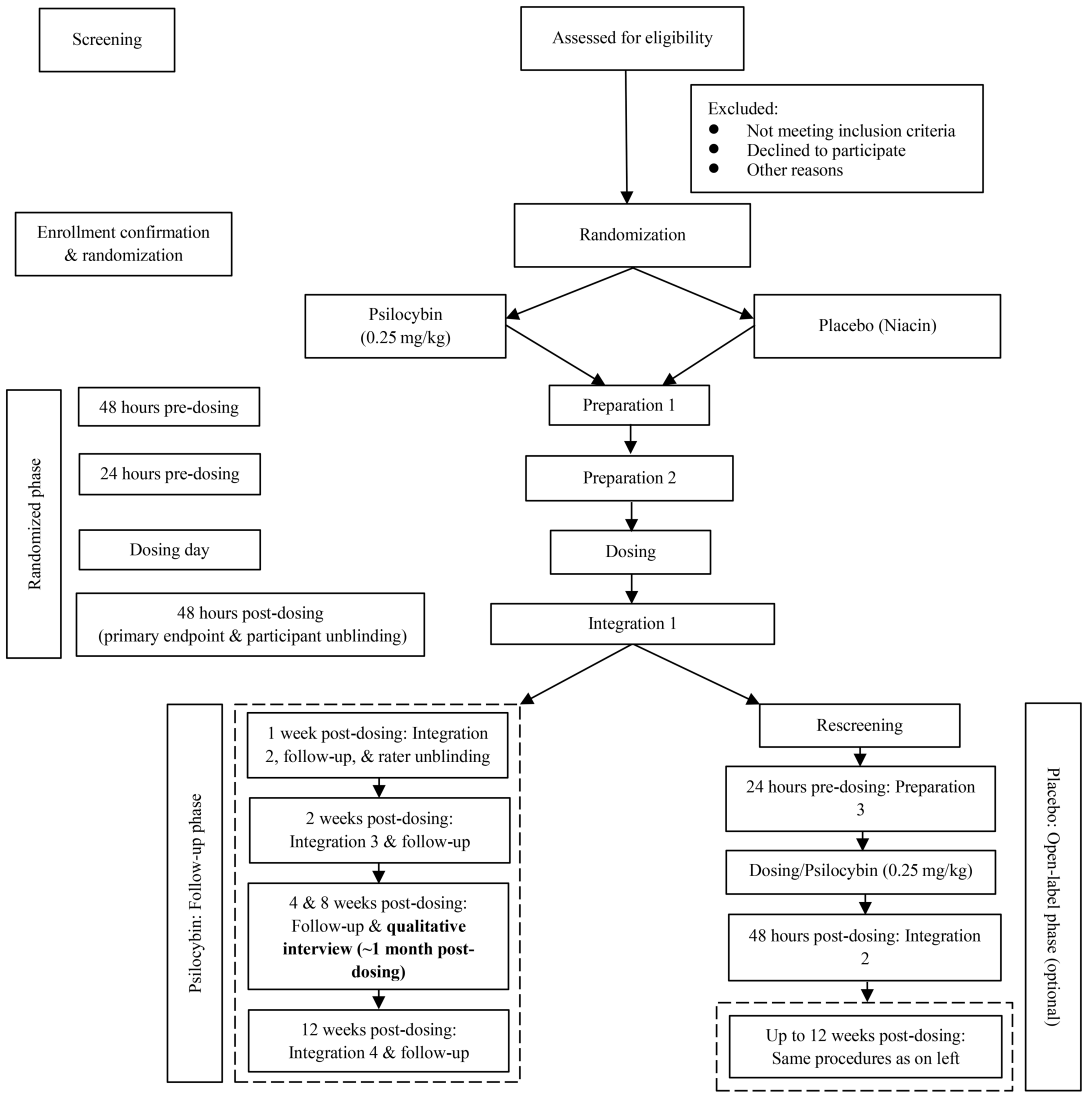
Simplified study flow diagram.

These studies signal the need for more rigorous controlled trials to elucidate the effects of psilocybin for OCD. In 2018, our group initiated a randomized, double-blind, niacin placebo-controlled trial examining the tolerability, safety, and efficacy of a single moderate dose (0.25 mg/kg) of psilocybin combined with unstructured and non-directive psychological support for participants with treatment-refractory OCD (31, 32). We also included an open-label arm for participants randomized to placebo to return for psilocybin dosing shortly after unblinding. **Figure 1** summarizes the design, structure, and flow of the trial. All participants had to (and were able to) abstain from any external treatments, including psilocybin, other medications, and psychotherapy, for the duration of the study. Our trial closed to enrollment in May 2024, and results indicated clinically significant mean reductions in OCD symptoms in the psilocybin group compared to placebo control at the primary endpoint of 48 hours post-dosing (33).

While quantitative measures provide standardized assessments of symptom change, they cannot fully capture the nuanced, subjective experiences that may explain *how* and *why* psilocybin impacts OCD, and vice versa. Qualitative patient perspectives fill this gap, and are an invaluable source of information for the advancement of ethical and rigorous psychedelic science in OCD and other clinical populations. Understanding how patients experience and make meaning of therapeutic changes is essential for optimizing treatment approaches. Therefore, we also conducted a qualitative interview with treatment completers approximately one month after either randomized or open-label psilocybin dosing.

Qualitative research in psilocybin trials offers several advantages. First, the textual data allows us to access complex phenomenological nuances of diverse – and sometimes idiosyncratic – psilocybin experiences that may be inadequately assessed on quantitative measures, especially in this understudied population. Through understanding how and why reported phenomena interrelate with one another, qualitative research allows us to develop a detailed and honest capture of participants’ experiences and realities before, during, and after psilocybin dosing. Second, qualitative research has the potential to uncover previously unidentified concepts of relevance to the phenomenology and effects of psilocybin dosing in OCD that may facilitate mechanistic hypothesis testing in follow-up studies. Thus, qualitative research represents an important data-driven, bottom-up approach to theory-building around psilocybin experiences in OCD. Third, discussions of hypothetical mechanisms resulting from qualitative inquiry and analysis contribute invaluably to the process of developing effective and personalized psychedelic treatments for OCD and other related disorders.

Therefore, in the present study, we were guided by the following overarching research questions in our qualitative inquiry: (1) What are possible prior influences on psilocybin experiences in OCD? (2) What are the acute effects of psilocybin dosing paired with psychological support for OCD? (3) What are the longer-term, post-dosing effects? (4) How do these prior influences and acute and longer-term effects interrelate?

## 2 Materials and methods

### 2.1 Participants

For this study, we focused our analysis on qualitative interviews conducted with a subsample of 12 treatment completers approximately one month after psilocybin dosing. We determined a-priori that 12 participants would provide sufficient depth for our analysis; data saturation was assessed iteratively, and new conceptual categories ceased emerging after the 10^th^ interview, with interviews 11–12 confirming saturation (see Section 2.3). These participants were the first 12 completers to consent to being interviewed and thus, constitute a convenience sample. Of these 12 interviewees, six received psilocybin in the initial randomized placebo-controlled phase, and six received open-label psilocybin following unblinding. **Table 1** displays each interviewee’s demographics, baseline OCD symptom severity as assessed on the Yale-Brown Obsessive-Compulsive Scale (Y-BOCS) (34, 35), primary OCD symptom dimensions, and psychiatric comorbidities. The purpose of our analysis is to detail subjective participant experiences, including but not limited to perceived effects on OCD symptom change. We refer interested readers to the primary outcomes paper (33) for reporting on quantitative study outcomes.

**Table 1:** Interviewees’ condition assignment, demographics, and clinical characteristics.

Participant ID	Randomized condition	Age	Gender	Race/ethnicity	Sexual orientation	Marital status	Baseline Y-BOCS score	Primary OCD symptoms	Other psychiatric diagnoses
3	Placebo	26	M	Non-Hispanic White	Heterosexual	Single	29	• Obsessive doubt, need to know/be certain, perfectionism, moral scrupulosity • Compulsive mental reviewing, indecision, reassurance-seeking and researching to establish certainty	GAD
5	Psilocybin	27	M	Non-Hispanic White	Heterosexual	Married	32	• Sexual and contamination-related obsessions, obsessive hyperawareness of chewing sounds • Mental compulsions, compulsive cleaning and washing, active avoidance of people who are eating	None
6	Psilocybin	38	M	Non-Hispanic White	Heterosexual	Married	24	• Obsessive doubt about being an imposter, primarily at work • Compulsively rigid and ritualized routines in daily activities, compulsive discarding and replacement of items to neutralize obsessive-self-doubt	MDD
8	Psilocybin	32	M	Non-Hispanic White	Heterosexual	Married	21	• Obsessions about letters and actions not feeling ‘just-right,’ obsessive fear of forgetting how to breathe • Compulsive rewriting and repeating of words and actions, somatic checking	MDD; panic disorder
9	Placebo	64	M	Non-Hispanic White	Heterosexual	Single	23	• Contamination-related obsessions • Compulsive, ritualized, time-consuming hygiene behaviors and somatic and mental checking for cleanliness	None
10	Placebo	35	M	Asian	Heterosexual	Single	25	• Obsessive hyperawareness of his swallowing, mental contamination, ‘not-just-right’ experiences • Compulsive swallowing and monitoring of his and others’ swallowing until ‘just-right,’ and rearranging and checking	None
12	Psilocybin	60	M	Non-Hispanic White	Heterosexual	In a committed relationship	22	• Harm-related, sexual, and other taboo obsessions • Compulsive self-reassurance, mental review, reassurance-seeking	None
13	Placebo	24	M	Non-Hispanic White	Heterosexual	Single	28	• Harm-related and sexual obsessions, obsessions about forgetting tasks or losing possessions, obsessive perfectionism in daily activities • Compulsive mental checking, checking of tasks, self-reassurance	MDD
15	Psilocybin	59	F	Non-Hispanic White	Heterosexual	Married	26	• Harm-related and relationship-focused obsessions • Compulsive reassurance-seeking, active avoidance of harm-related cues	BDD
16	Psilocybin	30	F	Non-Hispanic White	Heterosexual	Married	19	• Contamination- and harm-related obsessions, moral scrupulosity • Compulsive mental review, physical checking, reassurance-seeking	None
17	Placebo	23	F	Latina	Heterosexual	Single	23	• Contamination- and harm-related obsessions, ‘not-just-right’ experiences • Mental and physical checking, repeating routine activities until ‘just-right,’ reassurance-seeking	None
18	Placebo	22	M	Non-Hispanic White	Heterosexual	Single	28	• Obsessive perfectionism, ‘not-just-right’ experiences, moral scrupulosity • Mental and physical checking, repeating routine activities until ‘just-right,’ reassurance-seeking	None

Participants who were randomized to the placebo condition returned for open-label dosing after unblinding. M, cisgender man; W, cisgender woman. GAD, generalized anxiety disorder; MDD, major depressive disorder; BDD, body dysmorphic disorder.

### 2.2 Interview

A semi-structured interview was conducted for each participant approximately one month post-psilocybin dosing. This timepoint was selected to capture both acute memory of the experience and emerging longer-term effects, balancing recall accuracy with sufficient time for integration and symptom changes to manifest. Participants provided responses to questions anchored to aspects of their dosing experience, their experiences during the pre- and post-dosing phases, and their perspectives on therapeutic change. They were asked about their life leading up to enrolling in the study, their experience with OCD symptoms and the impact on their functioning, their treatment history, and their hopes, expectations, intentions, or goals for their time in the study. Participants were then asked about their dosing experiences, including but not limited to acute effects, emotions and memories that arose during the psilocybin dosing session, potential insights, realizations, or understandings, and any impact of OCD on their dosing experience (or vice versa). Items related to the post-dosing phase focused on the impact of psilocybin on OCD, insights about OCD after dosing, as well as any suggestions for future study modifications. The interview guide used for this study (see **Supplementary Material Section 1**) is similar to those used in prior qualitative studies of patient experiences with psilocybin for other indications (e.g., cancer-related anxiety, depression, smoking cessation, symptoms of posttraumatic stress disorder [PTSD]) (36–42).

The interviews lasted a mean of 98.3 min (SD = 19.72) per participant, ranging from 77 to 128 min. All participants were forthcoming and provided ample responses to each question, with typically little to no need for clarification or additional prompts by the interviewer.

### 2.3 Analytical approach

Utilizing interpretative phenomenological analysis (IPA) and the aforementioned research questions as a guide, we examined interviews to discern how participants made meaning of their psilocybin experience, seeking convergent themes across interviews (43). We selected IPA because it prioritizes participants’ lived experiences and meaning-making processes, which are particularly relevant given OCD’s ego-dystonic nature and the subjective quality of psychedelic experiences. IPA is a strategy for elaborating on interrelationships among participants’ reported thought processes, emotions, and behaviors, maximizing the richness of emergent themes. Further, IPA is a commonly used data analytical strategy in past qualitative studies of psychedelic experiences (36–38, 41).

**Figure 2:**
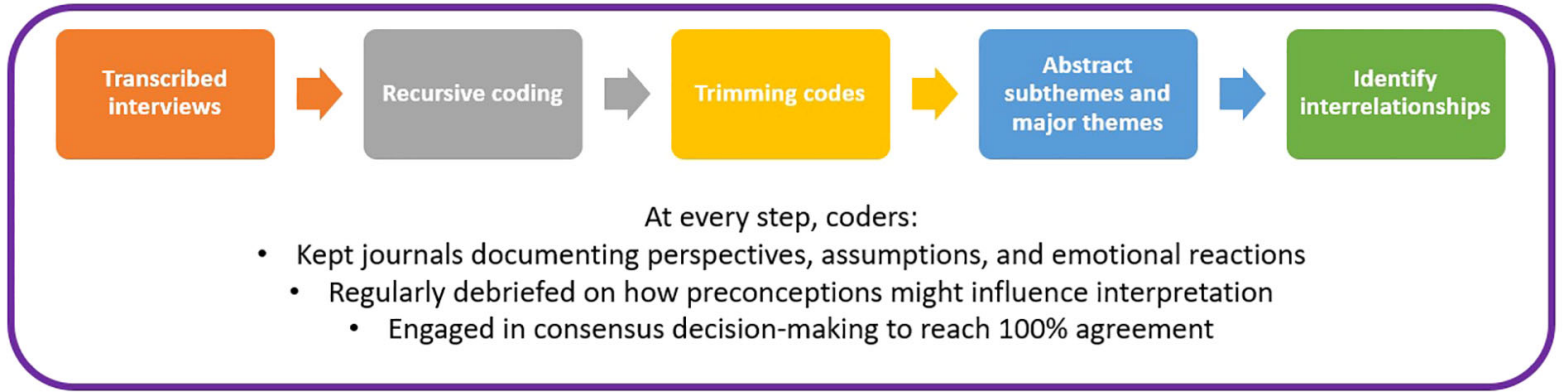
Summary of IPA process.

**Figure 2** summarizes steps in the IPA process for this study. The coding team comprised the first and second authors. To maintain reflexivity, both coders kept journals documenting their perspectives, assumptions, and emotional reactions during the analytical process. Regular debriefing sessions were held to examine how our backgrounds and preconceptions might influence interpretation. In terms of specific steps, the first author transcribed all interviews, read each line of each transcript, and created a preliminary working codebook, which was then revised during multiple subsequent rounds of independent, data-driven, and recursive coding alongside the second author. Codes were trimmed during this process; surviving codes pertained only to the timeframe of the research questions (i.e., only codes relevant to study duration, from enrollment to time of interview). Coders abstracted saturated codes into meaningfully organized higher-order subthemes and major themes. For something to be considered at least a subtheme, it needed to be present in at least half of the transcripts. Coders then collaboratively identified instances where codes, subthemes, and major themes may link together. Coders used a consensus decision-making approach (44) to reach 100% intercoder agreement at every phase of the analytical process. All IPA procedures were conducted using NVivo 14 software (45).

## 3 Results

**Figure 3:**
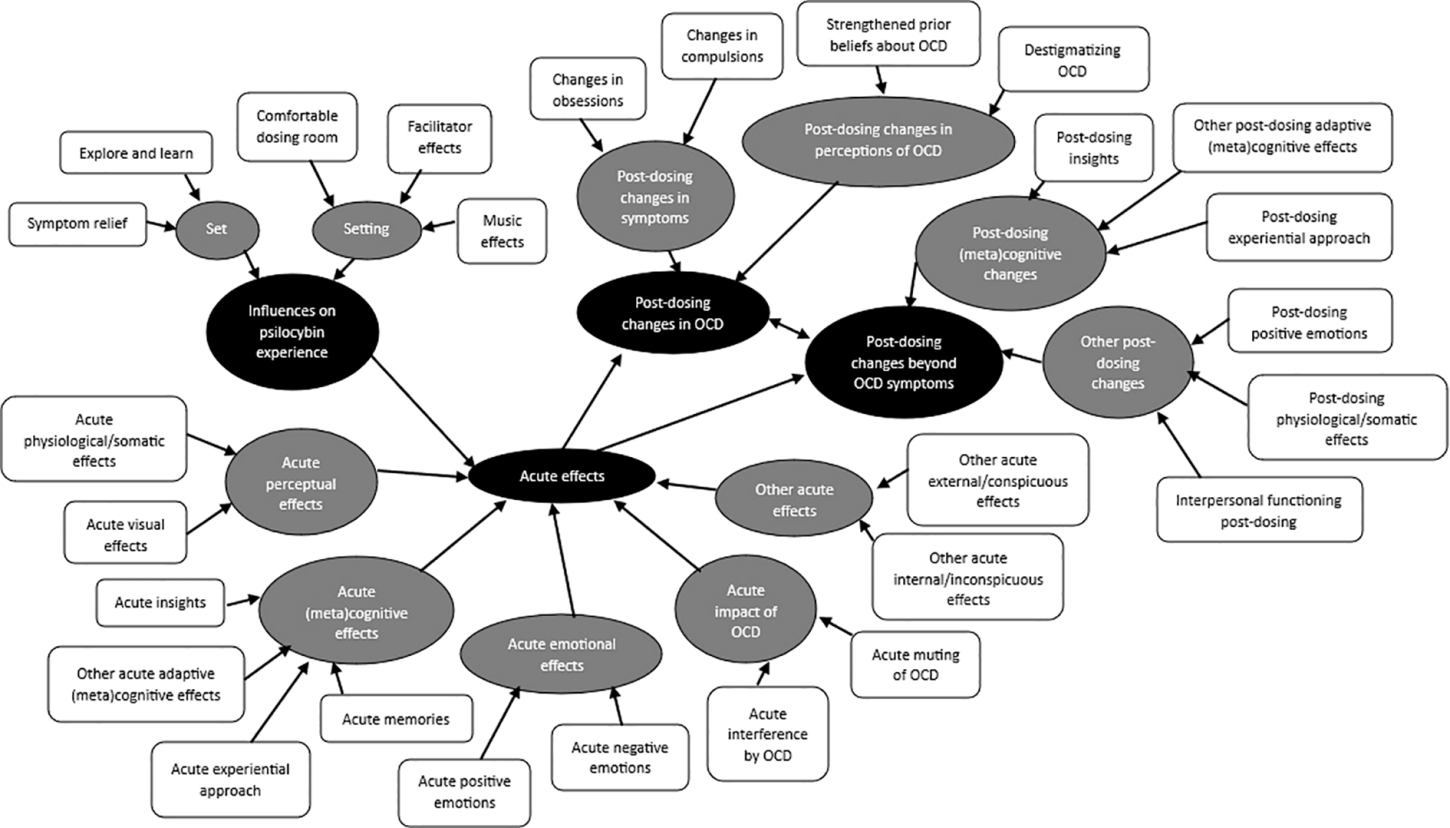
Major themes (black ovals), subthemes (gray ovals), and codes (white boxes) emergent from interview data. Interrelationships at all levels (i.e., within and between codes, subthemes, and themes) were the norm. For simplicity, we highlighted salient links with unidirectional and bidirectional arrows in this figure and detailed the rest in the text.

**Figure 3** illustrates emergent codes, subthemes, and major themes (i.e., Influences on Psilocybin Experience; Acute Effects; Post-Dosing Changes in OCD; Post-Dosing Changes Beyond OCD Symptoms) from our analysis, with interrelationships indicated by uni- and bidirectional arrows. In the following, we detail these emergent themes. **Supplementary Material Section 2** displays a fuller version of our results with representative verbatim participant quotes and concise interpretations.

### 3.1 Influences on psilocybin experience

#### 3.1.1 Set

##### 3.1.1.1 Symptom relief

While most participants expressed the intention of gaining some symptom relief through psilocybin, a minority expressed a desire for a cure. These participants adjusted their expectations more realistically in collaboration with facilitators during preparatory sessions, which helped shape more fruitful engagement in subsequent sessions.

##### 3.1.1.2 Explore and learn

Additionally, participants generally expressed the intention of being open to arising experiences during dosing. They wanted to explore and learn new things about their OCD, how to manage it, and even learn more about themselves beyond their OCD.

#### 3.1.2 Setting

##### 3.1.2.1 Comfortable dosing room

Participants described feeling comfortable in the dosing room due to the level of attention devoted to the décor by the study team. Participants also reported ease in proceeding with the dosing session due to dosing room adjustments accommodated by the study team. Participants who received placebo first also cited increased familiarity with the room during the open-label phase, which made it easier for them to focus on their intention for the dosing session, and immerse themselves into the experience.

##### 3.1.2.2 Facilitator effects

Facilitators’ non-directive, highly attuned, supportive approach helped increase trust, rapport, and comfort during dosing, and was perceived as grounding during distressing or challenging moments for participants. Facilitators also helped shape some of participants’ acute experiences, or otherwise assisted them in navigating or immersing in these experiences through supportive touch (e.g., hand holding) and attuned, intuitive, non-directive prompts.

Additionally, facilitators supported participants in arriving at various insights (e.g., about and beyond OCD) during dosing by acting as a ‘sounding board,’ and were often the proximal target of acute positive emotions during dosing (e.g., participants feeling connected or bonded to facilitators). Further, participants generally appraised their interactions with facilitators as positive and helpful during integration sessions, because facilitators continued to support participants in building upon insights from dosing.

##### 3.1.2.3 Music effects

Participants generally perceived the dosing session music playlist as calming and pleasant, leading to vivid visualizations, or interacting with the psilocybin experience in ways that positively enhanced other acute effects. The playlist was designed to mimic the prototypical arc of the psilocybin experience; the exact track listing can be found on page 57 of the facilitator manual (46) for a current, separate psilocybin-OCD trial that our research group is conducting (47). Therefore, most participants unsurprisingly cohered on the music syncing with the onset of peak effects. Most participants also viewed the music as emotionally evocative or facilitative of catharsis at parts.

There were, however, participants who complained that parts of the music, especially those with vocalizations in a foreign language, were overwhelming and intrusive. The observations highlighted the influence of one’s expectations about what dosing experience music should sound like.

### 3.2 Acute effects

#### 3.2.1 Acute perceptual effects

##### 3.2.1.1 Acute physiological/somatic effects

Participants reported diverse acute physiological changes and somatic sensations. Some of these were positive: pleasant tingling sensations (Participant 16); full-body relaxation (Participants 5, 12, 13); embodied lightness or unburdening of a psychosomatic weight (Participants 3, 10, 18); and realignment with one’s body (Participants 5 and 8). These effects were accompanied by congruent emotions, such as feelings of safety, calmness, gratitude, hopefulness, joy, excitement, and bright-eyed curiosity and awe about one’s body and the world.

Additionally, in a few of these instances, visual imagery was also an integral component of positively experienced physiological and somatic effects, such as visualizing oneself submerged in a warm ocean while experiencing physical relaxation (Participant 13), visualizing white energy emanating from one’s chest with a felt sense of physical unburdening (Participant 10), or visualizing oneself as a tree or in the midst of rebirth while feeling more grounded and rooted in one’s body (Participant 8).

In other instances, participants experienced discomforting numbness, pressure, or sensations on different body parts (Participants 6 and 10), or uncomfortable tactile changes (e.g., “cottonmouth;” Participant 17), accompanied by negative emotions. Some of these experiences were also familiar to participants. For instance, Participant 16 described a deep, weighted, fully embodied experience of sadness akin to her reaction to the death of her father.

For a minority of participants, positive acute physiological and somatic effects allowed them to reach novel understandings about the importance of their body in their day-to-day functioning. For example, Participant 18 described pleasant feelings of physical relaxation as precipitating a new somatic awareness, one that allowed him to fully get in touch with his body, which contributed to the insight about the importance of taking care of his body through intentional self-care practices.

##### 3.2.1.2 Acute visual effects

Participants experienced a vast array of mostly closed-eye imagery (e.g., geometric, kaleidoscopic patterns), as well as illusions of objects in their visual field, some of which had a synesthetic quality. These tended to aligned with commonly reported visual perceptual changes with psilocybin.

For some participants, visual effects were closely tied to acute insights about their OCD. For example, Participant 10 saw a white light grow brighter (visual effect) while his eyes were closed, as he approached the “ultimate personal truth of [his] OCD” (insight). This synergy appeared to amplify the emotional impact and memorability of the insight. Participant 17 described another profound visual experience of different paths in life she could take in spite of frequently feeling disempowered by her OCD symptoms.

Some of these visualizations were also vivid recreations of significant past memories, during which participants were either able to achieve resolution about past events (e.g., having a corrective visualization of a childhood memory with an alcoholic father; Participant 15), or reawaken dormant positive feelings (e.g., feelings of safety as “a child in a pillow fort;” Participant 13), which were then accompanied by deeper understandings about these past events or current needs.

Additionally, some visualizations manifested as idealized relationships with loved ones, which prompted realizations of how important these relationships were to them, in spite of past or ongoing conflict (e.g., visualizing oneself on death bed accompanied by parents and feeling their love; Participant 3).

Overall, visual effects elicited mixed emotions. For some, spontaneous closed-eye nature-related or fantastical imagery, as well as visualizations of loved ones, elicited feelings of calmness, love, gratitude, joy, safety, warmth, comfort, and connectedness. In one instance, Participant 6 reported feelings of confidence about navigating the remainder of the psilocybin experience after overcoming a “sea storm” of challenging effects.

On the other hand, other specific imagery accompanied, or perhaps were the visual manifestation of, arising negative emotions. Participant 10 reported experiencing closed-eye imagery of looping, winding paths while feeling stuck at moments of his psilocybin journey, which was accompanied by significant emotional discomfort. Participant 17 described confusing and fear-inducing visual illusions of seeing his facilitators’ faces “melt and shift.” Additionally, Participant 3 encountered a contamination-related trigger (uncertain whether his lips touched an open bottle of essential oils), accompanied by striking visual imagery (poison entering his mouth and body) and feelings of fear, anxiety, and uncertainty.

#### 3.2.2 Acute (meta)cognitive effects

##### 3.2.2.1 Acute insights

Participants reported experiencing various insights during dosing, including some about the etiology or true nature of their OCD. Participants 3, 5, 10, and 15 mused upon unexplored links between stressful early-life experiences and past coping/control strategies, as well as between OCD onset and persisting, thematically relevant symptom content. Participant 5 described realizing that OCD is a “rigid definer” that attempts to “figure out things that are impossible to define.” Further, pt 16 provided a normalizing perspective on her OCD, coming to the realization that “OCD might not be this malicious force [after all].”

Participants also described acute insights about the possibility of a more adaptive relationship with their OCD. Participant 10 shared that he felt like he could “let go” of his OCD. Participant 9 described realizing that he actually had autonomy over his OCD, including “letting go” and “going with” arising experiences, and making necessary changes to address his symptoms. Participant 15 also reported “making peace” with the possibility that OCD will persist for her, given that there are “a lot of things in [her] life that [she] can be grateful for;” these things can “coexist.”

Other acute insights related to participants’ potential for more adaptive psychological processes in general. Participant 12 described realizing that “things aren’t always black-and-white,” while Participant 18 understood that negative thoughts and emotions are “just that – thoughts and emotions.” Participants 8, 12, and 15 also converged on possibility of a full inner life in spite of negative internal events.

Acute insights also involved the importance of devoting more attention to self-care (Participants 13 and 18). Others were relational and retrospective; Participant 15 realized that her father loved her in spite of his alcoholic outbursts, while Participant 16 reckoned with the gravity of stressors in her early life and her understandable need to grieve the childhood she lost. Participants 8, 10, and 13 believed that they came in touch with “universal truth,” to which they surrendered, to varying extents.

In terms of intersecting codes, participants described insights co-occurring with visual imagery (see Acute visual effects subsection) and positive emotions. In the latter, participants described feeling a deepened sense of connectedness (8, 10, 13), intense feelings of love (3, 15), gratitude (15), and self-compassion (16). Participant 18 also described feelings of optimism accompanying the acute belief that he could successfully manage his OCD moving forward.

Notably, participants’ insightful moments co-occurred with other adaptive (meta)cognitive effects that represented a novel stance towards difficult internal experiences (e.g., obsessions, negative emotions, negative automatic thoughts, or dysfunctional core beliefs). Psilocybin arguably facilitated such pivotal states of mind and insights, both of which appeared to be tightly intertwined. We discuss these adaptive (meta)cognitive effects next.

##### 3.2.2.2 Other acute adaptive (meta)cognitive effects

Most participants reported experiencing various acute adaptive (meta)cognitive effects, some of which map onto mechanistic targets (e.g., decentering, mindfulness, cognitive defusion, self as context) in third-wave mental health treatments such as acceptance and commitment therapy (ACT) (46).

Participant 10 described psilocybin facilitating a state of mind where his OCD was suspended, allowing him to tap into his inner wisdom about the importance of “letting go” of OCD. Participants 3 and 5 recalled a more decentered awareness of OCD (e.g., experiencing the loosening of “OCD’s grip” on cognitive processes).

For other participants, psilocybin facilitated a mentally expansive, mindful state. Participant 12 described holding all difficult thoughts and emotions – including obsessions and compulsive urges – in his mindscape, akin to the ACT process of self as context (48). Participant 16 recalled the ability to defuse from negative inner experiences (e.g., telling OCD that she “[does] not need to figure things out”). In doing so, participants were capable of welcoming or redirecting attention to concurrent, positive unfolding experiences, such as Participant 15’s gratitude for others in her life, Participant 12’s appreciation of the beauty in pictures of family members or how fruit tasted, and Participant 13’s admiration of particular room décor with bright-eyed wonder.

In terms of intersecting acute effects, as participants reached pivotal (meta)cognitive states, they also often experienced intense positive emotions. Moreover, participants’ adaptive (meta)cognitive experiences were also examples of experiential approach (see next section), or the process of flexibly ‘going with’ arising experiences or ‘letting them be,’ instead of rigidly controlling, eliminating, or avoiding such experiences (i.e., experiential avoidance) (49–52). For instance, Participant 12 recounted an example of continuing to approach/appreciate a picture of his sister, a source of his taboo obsessions, rather than engaging in his usual avoidance or mental compulsions.

##### 3.2.2.3 Acute experiential approach

As mentioned, participants reported idiosyncratic instances of experiential approach in the face of challenging arising events. The act of letting go of the need to control and ‘being with’ arising negative experiences paradoxically led participants (e.g., Participant 17) to appreciate the calmness that came with accepting, rather than struggling against, these transient events.

Other instances interacted with participants’ acute obsessions. Participant 3 explained how psilocybin-occasioned feelings of losing control activated his obsessions, which prompted him to confront his core fears with his facilitators’ help, leading to a powerful moment of learning (e.g., that he can take risks in spite of obsessive fears).

Experiential approach also occurred during participants’ acute recall of significant, traumatic memories. Notably, Participant 8 reported surrendering to and gaining clarity about a typically avoided memory (i.e., being part of a group of students that sent in an anonymous tip at his school that a student was in possession of illicit drugs; that same student subsequently committed suicide after being expelled).

##### 3.2.2.4 Acute memories

Participants’ acute memories co-occurred with positive emotions, and tended to be precipitated by acute visual perceptual changes. Participant 12 described a spontaneous, closed-eye visualization (e.g., wholesome, surprising imagery of entering a tunnel or series of vaginas), which prompted fond memories of women in his life. Additionally, Participant 13 described having a vision of building a pillow fort in an unfamiliar living room, which prompted memories and feelings of safety as a child, experiences that were hard to access for him as an adult. Participant 15 also shared a corrective experience of learning how to drive as her teenage self with her father who had passed, which arose out of a similar spontaneous memory. Lastly, Participant 9 experienced a vivid visualization of a drafting table, which he recognized as being the same one his father had worked on his workshop before he passed. While speculative, it is possible that these memories arose from dormancy due to the extensive exploration of participants’ family and psychosocial histories during preparatory sessions.

#### 3.2.3 Acute emotional effects

##### 3.2.3.1 Acute positive emotions

As mentioned in earlier sections, participants experienced diverse, acute positive emotions, whether in itself, towards others, towards themselves, or as a result of interactions with facilitators or other aspects of the psilocybin experience or setting. These emotions largely cohered with previous observations in other psilocybin studies (36–42).

These emotions included: bliss (Participant 10); self-compassion for current or younger selves (Participants 3, 10, 15, 18) and compassion towards OCD as part of their being (Participant 16); and love and gratitude for others and the things they have in life (Participants 3, 6, 12, 15). Other emotions included: self-confidence (Participants 6, 13); feeling safe, secure, and protected (Participants 13, 15); connectedness and closeness to friends, family, and facilitators (Participants 8, 13); joy, happiness, and euphoria (Participants 5, 8, 13, 16, 18); calmness (Participant 17); hope and excitement for the future (Participant 18); trust in self (Participant 13) and in facilitators (Participants 3, 8); bright-eyed wonder (Participant 13) or awe in the felt presence of beauty (Participant 5); as well as feelings of resolution about difficult past events (Participant 6).

Participants did not experience acute positive emotions in isolation; commonly, these occurred in conjunction with, or as the result of, acute insights, perceptual changes, and other adaptive (meta)cognitive effects, as detailed in earlier sections. Participant 15 reported seeing her 7-year-old self, and simultaneously experiencing deep sympathy for herself in that moment. Participant 10 provided another example of blissful synesthesia (e.g., colors streaming across his mind to indigenous music).

Further, positive emotions co-existed with negative emotions (see next section). Oftentimes, participants experienced emotions across the spectrum concurrently, volatilely, and cathartically.

##### 3.2.3.2 Acute negative emotions

Participants reported experiencing various acute negative emotions, such as distress, irritation, anger, fear, doubt, and/or anxiety/panic, for instance, when OCD intruded upon their psilocybin experience (Participants 3, 15), when disturbed by visualizations (Participants 6, 10, 17), when misinterpreted by a facilitator (Participant 12), or in anticipation of further acute effects (Participant 18). Additionally, participants reported experiencing sadness, grief, frustration, emotional pain, feelings of loneliness, shame, and/or guilt, for example, when revisiting painful past memories/events (Participants 15, 16), reckoning with interpersonal disconnection (Participant 17), or realizing that the psilocybin session was winding down (Participant 5).

Negative emotions were not experienced in a vacuum; such moments co-occurred with acute positive emotions, insights, and visual and somatic perceptual changes (e.g., see Acute physiological/somatic effects subsection for Participant 16’s example).

#### 3.2.4 Acute impact of OCD

##### 3.2.4.1 Acute interference by OCD

OCD intruded on several participants’ psilocybin experience; obsessive fears were activated in anticipation of, during, or after the onset of aforementioned acute effects (e.g., Participant 3’s visualization of poison entering his body as a manifestation of contamination fears). In a subset of participants, such obsessions were accompanied by compulsions in the attempt to alleviate activated fears and anxiety. Such occurrences were also coded as instances of experiential avoidance. For instance, Participant 17 described frequently taking off the headphones and eyeshades to “step out” of her experience as an example of her typical avoidance and compulsive neutralization behaviors.

##### 3.2.4.2 Acute muting of OCD

A minority of participants reported the absence of OCD symptoms during dosing, with psilocybin ‘turning off’ or muting their OCD. Participant 5 described how the “definer” that was the OCD was not active during part of his dosing experience, while Participant 15 speculated on psilocybin’s acute surgical effects on her OCD (i.e., having the “real sense” that something was “taken out” of her brain, and “hoping it was the OCD”).

It was, however, more common for participants (Participants 3, 12, 13, 16, 18) to ease into letting go of the need to respond to arising obsessive fears for part of their psilocybin session, while attending to other pleasurable aspects of their psilocybin experience (e.g., see Other acute adaptive [meta]cognitive effects section for Participant 12’s example). Additionally, participants generally oscillated between engaging with and disengaging from OCD symptoms during dosing. Furthermore, for Participants 3 and 9, the presence and support of facilitators were integral in their ability to navigate arising OCD symptoms without excessive maladaptive reactions (e.g., compulsions, panic).

#### 3.2.5 Other acute effects

##### 3.2.5.1 Other acute internal/inconspicuous effects

Participants experienced unique acute internal effects that were not adequately captured within aforementioned categories. Participants 13 and 17 experienced feelings of universal oneness and possible encounters with “ultimate reality.” Participants 10 and 15 reported encounters with their younger selves, while Participants 5 and 17 described moments of childlike impressionability. Participant 13 also described intense feelings of artistic creativity and a yearning for new life experiences. There was also a trend of altered time perception; specifically, feeling like more time has passed than actual. Lastly, Participant 17 reported immersion in unfolding experiences and temporary disorientation to place and presence of facilitators, while Participant 8 described self-convincing anthropomorphic changes (e.g., disappearing into a black hole; collapsing unto himself until he was the size of a nucleus; decomposing into soil; becoming a tree).

##### 3.2.5.2 Other acute external/conspicuous effects

A smattering of other acute external effects was also reported, primarily involving cathartic expressions during evocative or challenging moments. Participants 3, 5, 16, and 17 described crying to express, process, or expel pent-up emotions, while Participant 6 focused on squeezing out pain, discomfort, and negative feelings out of his left leg.

### 3.3 Post-dosing changes in OCD

#### 3.3.1 Post-dosing changes in symptoms

##### 3.3.1.1 Changes in obsessions

Participants reported variable improvements in obsessions after dosing. For example, Participant 10’s primary obsessive hyperawareness persisted, while his secondary obsessions abated. More commonly, for others (Participants 3, 5, 9, 12, 18), their relationship with their obsessions evolved in positive ways in spite of persisting obsessional severity and content (see sections below for more). For a minority (Participants 13, 16, 17), total symptom relief was brief, with obsessions returning the days after dosing. Yet others (Participants 6, 8) described full remission of obsessions, a stark change from before dosing. Therefore, heterogenous trajectories were apparent.

Notably, participants (e.g., Participant 3) viewed non-directive support from facilitators during dosing and integration as integral to perceived improvements in obsessions. Participants 6 and 8 also explicitly attributed symptom remission to the myriad of acute effects (e.g., death and rebirth experiences) they experienced during dosing, which culminated in profound insights and shifts in priorities.

##### 3.3.1.2 Changes in compulsions

As expected, given the functional relationship between obsessions and compulsions, participants also reported variable improvements in compulsions. Some (Participants 6, 8) reported remission in compulsive behaviors stemming from acute psilocybin effects, while others (Participants 13, 16, 17) reported no change. More commonly, participants (Participants 3, 5, 9, 10, 12, 18) experienced noticeable reductions in compulsions (e.g., touching things that were previously avoided due to contamination fears) as a result of adaptive shifts in their relationship with their OCD symptoms (see sections on changes beyond OCD below).

#### 3.3.2 Post-dosing changes in perceptions of OCD

##### 3.3.2.1 Strengthened prior beliefs about OCD

A subtheme that emerged in the post-dosing phase was a shift in perceptions of one’s symptoms. For several participants, their overall psilocybin experience seemed to strengthen their pre-existing beliefs about their symptoms.

Participant 16 described how an acute experience of her OCD as a natural part of her physiology during psilocybin reinforced her predominantly neurobiological/genetic causal attribution for her OCD. Other participants described strengthened psychological attributions for their OCD. Participant 12 described how negative emotions exacerbated his OCD, while positive, prosocial emotions were effective counterpoints. Participant 15 believed that her OCD was a coping mechanism for her father’s problematic drinking, like how OCD is possibly a “coping mechanism” for stressors in people’s lives. Likewise, Participant 10 stated that OCD was an unhealthy coping mechanism for anger about things outside of his control, especially in his childhood.

Yet others (Participants 8, 13) expressed strengthened belief in a diathesis-stress explanation of their OCD symptoms. Participant 8 shared the deepened conviction that his OCD was the outcome of his “neurochemical predisposition” and his “learning environment,” in which OCD-related behaviors were “normalized” by his father, whom also suffered from OCD.

Interestingly, Participant 17 expressed non-ontological beliefs about his OCD post-dosing; he reiterated that psilocybin reinforced his pre-existing ideas about “how to live and manage OCD in a more adaptive way.”

##### 3.3.2.2 Destigmatizing OCD

Participants’ OCD-related shame and self-stigma dissipated post-dosing, which appeared to be tied to their changing relationship with their persisting, residual, or remitted symptoms. Although Participant 3’s symptoms were “still noticeable” post-dosing, he managed to arrive at a healthier attitude toward his OCD, recognizing it as an intrinsic quality that he can “work on” to “make better.”

Participants 8 and 9 also described decreased stigma and concealment and increased acceptance and disclosure of their OCD due to noticeable symptom attenuation. Participant 8 expressed confidence in being able to recognize personal signs and symptoms of relapse. Participant 9 elaborated how his family shifted from rejecting to welcoming his disclosure since his improvement in symptoms, which increased his willingness to share further details of his inner life (see section on post-dosing changes in interpersonal functioning below).

### 3.4 Post-dosing changes beyond OCD symptoms

#### 3.4.1 Post-dosing (meta)cognitive changes

##### 3.4.1.1 Post-dosing insights

A variety of post-dosing insights emerged, either persisting from dosing or forming spontaneously or during integration sessions with facilitators. These insights meaningfully clustered around four foci.

First, some post-dosing insights concerned OCD specifically. Participant 5 described a unique insight during integration sessions that his OCD may be maintained by his “disciplined” treatment of his body. He described the possibility that because he “only respond[s] to hunger or anxiety,” his body would send excessive anxiety signals (i.e., obsessions) to prompt him to attend to his other needs. Additionally, Participant 17 realized during dosing that her OCD “very much liked to be in control of things.” She also credited psilocybin for the insight post-dosing that her compulsions “do not serve a meaningful purpose.”

Second, some post-dosing insights converged on how to live with (or without) OCD. Participant 3’s attempts at experiential approach during dosing were empowering, prompting persisting insights about taking appropriate risks and letting go of the need for control in regards to his OCD. Participants 3 and 10 also expressed interest in ACT (for Participant 3, especially the principles of acceptance and valued action), which aligned with their insights. Participant 17 expressed deepening post-dosing insights about how her anxiety-driven avoidance behaviors paradoxically maintain her OCD. Similar to Participants 3 and 10, she also expressed interest in resuming therapy and/or medications for her OCD, citing living with OCD as a “work-in-progress.” Additionally, Participant 8 believed that his experience of “dying” in his psilocybin session was integral to truly understanding that approaching his fears was the way to maintain remission from OCD, because he had confronted a major human fear (i.e., death) and “was okay.” Further, Participant 18 expressed the profound insight post-dosing that “happiness comes when [he is] not trying to be perfect,” contrary to the perfectionistic nature of his OCD.

Third, some post-dosing insights concerned aspects of participants’ lives outside of OCD concerns. Participant 15 stated that the study has helped her realize that she has “a lot to be thankful for in life,” such as her family and friends. Participant 6 also described unfolding shifts in his life priorities, such as nurturing and spending time with his daughter, which he “would not trade anything for.”

Fourth, a few post-dosing insights were reverent of psilocybin’s therapeutic effects. Participant 3 described psilocybin as a “profound teacher” that “broke [him] out of [his] obsessions” and allowed him to tap into his ability to let go of the need to control, take risks, and tolerate distress, all of which he attributed symptom reduction to. Participant 8 also described psilocybin as a catalyst to his post-dosing gains.

Lastly, these insights tended to be tied to altered perceptions of OCD (see section above) and co-occurring post-dosing (meta)cognitive changes (see next).

##### 3.4.1.2 Other post-dosing adaptive (meta)cognitive effects

Several participants described adaptive (meta-)cognitive processes post-dosing that clustered into two categories.

In the first category, participants appeared to pivot from a self-defeating to a self-efficacious or resilient outlook on life, in spite of OCD. These shifts were also accompanied by a variety of positive emotions.

For instance, Participant 3 experienced a growing ability to accept – and even love – OCD as part of his identity. As such, he reported renewed self-efficacy to manage his OCD through self-exposures, reappraising feelings of anxiety as “a sign” that he is taking difficult but necessary steps to manage his OCD. Participant 9 expressed post-dosing agency and self-efficacy over OCD symptoms, accompanied by feelings of optimism and confidence. Participant 12 expressed an optimism for life in spite of ongoing OCD symptoms, and felt more equipped to navigate interpersonal conflicts, due to an increased ability to disengage from an OCD-related mode of “hyper-criticalness” and antagonism.

Post-dosing, Participant 13 shifted from rumination about his past to a healthier perspective on life and his future. Participant 15 believed that the study helped her “discover [her] inner strengths,” enabling a sense of resilience about future OCD-related challenges. Lastly, Participant 18 reported feeling more aligned with his true self and values the day after dosing. At the time of interview, he endorsed self-efficacy and autonomy of choice in enacting behaviors consistent with his values, instead of as dictated by OCD.

In the second category, participants reported having generally more flexible relationships with challenging inner experiences, OCD-related or not.

For instance, Participants 16, 17, and 18 reported a more accepting attitude about the reality of living with OCD and a reduction in inflexible thinking. Participants also described being more able to hold the dialectic about opposing mental experiences. Participant 3 explained how even though he continues to have intrusive thoughts, he recognizes them as obsessive in nature and is able to dismiss them. His perceptions of his psilocybin session as challenging *and* a source of great learning about himself also catalyzed his post-dosing ability to embrace the dialectic for most things in life; this appeared to be a shift from his previous mental rigidity.

Participant 5 described experiencing defusion (or, a sense of separating from) and autonomy over OCD post-dosing, alongside enhanced distress tolerance. He attributed these changes to the insight during dosing that his OCD is “not a disease,” but rather, “a part of how [his] brain works.” Participant 6 also described recognizing post-dosing that his mental experiences are mere objects in his consciousness, a perspective that persisted from dosing. Lastly, Participant 8 reported increased disengagement from his thoughts, which represented a change from his previous obsessive state. He concluded that his thought content was not problematic; rather, he viewed his mental and behavioral responses (i.e., obsessions, compulsions, worries) as being the source of his previous distress. Participants 9, 10, 12, and 15 also described an improved ability to disengage from their obsessions.

##### 3.4.1.3 Post-dosing experiential approach

Post-dosing, the majority of participants described approaching, to varying extents, internal or external events that would previously prompt avoidance. In most of these cases, participants approach with a sense of neutrality or reduced consequentiality, rather than resorting to their previous avoidance or compulsive patterns.

Participant 17 reported approaching lower-stakes triggers of her OCD and anxiety/panic symptoms, while continuing to avoid other more anxiety-provoking stimuli. Participant 3 recognized the need to push himself out of his comfort zone (e.g., resisting anti-anxiety medications even when anxious), attributing this change to his decision to “take a leap of faith” and “let go of the need to control” during dosing. Participant 5 described the ability to “let [obsessive thoughts] be” rather than respond with avoidance or compulsions, citing the dosing-related insight that OCD symptoms are just “a part of how [his] brain works.”

Participant 6 explained that his OCD symptoms were improving due to the newfound ability to “let go” of the need to “figure things out,” in spite of ongoing urges. Participant 8 also recounted how his metamorphic experiences during dosing has inspired him to adopt a mindful stance toward negative (and positive) thoughts and emotions, “letting go” of the learned need to struggle against internal experiences. Further, Participant 9 described attempting to recreate his dosing experiences of ‘going along or flowing with’ perceptual changes, albeit with daily activities, instead of “being [as] on guard” as his OCD demands.

Notably, Participant 18, described enacting his post-dosing insight and value of living more fully in spite of OCD, specifically, by being spontaneous and not giving in to perfectionism-related obsessions and compulsive urges (i.e., to reread and rewrite).

#### 3.4.2 Other post-dosing changes

##### 3.4.2.1 Post-dosing positive emotions

Participants reported diverse positive emotions post-dosing; some feelings tapered off in the month prior to the interview, while others were still salient at the time of the interview. These emotions were strongly related to persisting insights, adaptive (meta-)cognitive and interpersonal changes, and reductions in OCD symptoms.

These included: well-being (Participant 10); pure joy (Participants 9, 10); feeling carefree (Participants 6, 9); feelings of inner peace and calmness (Participants 5, 9); love, acceptance, and compassion toward self, OCD, and others (Participants 3, 5, 8, 9, 10, 13, 18); gratitude for the study and other people and life experiences (Participants 3, 10); as prosocial feelings toward friends and family (Participants 3, 6, 8, 15); excitement and optimism about living with OCD (Participants 12, 13, 18); and hope for psilocybin as a breakthrough therapy for OCD (Participants 12, 16).

##### 3.4.2.2 Post-dosing physiological/somatic effects

Several participants reported physiological or somatic effects the evening and morning after dosing, all of which dissipated within 24 hours. Negative effects included fatigue, headache, hunger, and feeling hung over, while positive effects included feeling physically and mentally lighter, and restful sleep.

##### 3.4.2.3 Interpersonal functioning post-dosing

Post-dosing, Participants 3, 9, 10, 12, and 17 reported increased interpersonal connectedness, including talking to friends and family more about their OCD and/or psilocybin experience, or engaging in shared activities. To illustrate, Participant 6 reported prioritizing spending time with his daughter over any career opportunities (e.g., becoming vice-president of the company he works for). Also, Participant 8 explained how with the remission of his OCD, he was now more equipped to reciprocate his wife’s caregiving and support over the years, and ease her burden from their son’s ongoing medical issues.

## 4 Discussion

In this study, we conducted a qualitative analysis of post-dosing interview data from 12 completers in the first, randomized, double-blind, single-dose, placebo-controlled trial of psilocybin for OCD (31, 33). While distinct themes emerged, interrelationships among codes, subthemes, and themes were the norm.

Our findings regarding pre-dosing factors highlight the moderate to strong influence of extra-pharmacological factors of set and setting (53, 54) in the nature and trajectory of participants’ psilocybin experiences. Our approach to psychological support in this trial was unstructured and non-directive, to provide monitoring of participants’ psychological and physical safety while emphasizing their autonomy, agency, and power to chart their own path in their psilocybin journey (32). Unsurprisingly, despite our best intentions and overall fidelity to the model of support, music and facilitator effects had a considerable impact on participants’ psilocybin experience. The playlist contained evocative music that helped shape certain participants’ reported experiences (55, 56). Additionally, facilitator support, in the form of words of encouragement, reassurance, active listening, or brief supportive touch, was instrumental at times for certain participants to arrive at key insights regarding their psilocybin experience (57–59). These findings demonstrate that psilocybin effects do not occur in an intraindividual vacuum, but rather is subjected to complex, synergistic interactions with elements outside of the self and the drug (60, 61).

Where acute psilocybin effects are concerned, we uncovered several visual/perceptual, emotional/psychological, and physiological/somatic effects that map onto those commonly reported in psilocybin clinical trials for other closely related indications (e.g., body dysmorphic disorder, depression, illness-related anxiety) (26–28), as well as in healthy control studies (22, 62). Our findings speak to transdiagnostic manifestation of acute psilocybin effects, and perhaps more so, to the interactive, synergistic nature of such effects in creating a multisensory, multifaceted dosing experience.

At the same time, perhaps owing to dosage, setting, population and disorder characteristics, and other protocol or methodological differences, in our trial, participants rarely described the full array of acute effects that together prototypically constitute ‘full mystical experiences’ described in landmark studies (e.g., psilocybin for existential distress or depression) (21, 63). While debate persists on what defines a mystical psychedelic experience, the following aspects are likely robust characteristics: state of transcendence and interconnectedness, profound emotionality, feelings of reverence of sacredness tied to having had a psychedelic experience, experiences of enlightenment about salient or emergent topics, the holding of paradoxical truths and realities, and ineffability regarding these experiences (64, 65). Our participants with OCD generally described a narrower range and/or intensity of these acute effects, making for what may be more accurately described as ‘partial mystical experiences.’

From our analysis, the emergence of OCD symptoms during dosing (e.g., obsessions or compulsive rituals overtaking or distracting from the unfolding psilocybin effects, or being heightened due to such effects) likely hindered immersion in the psychedelic experience, thereby possibly accounting for such ‘partial mystical experiences.’ Indeed, ‘partial’ experiences were the source of disappointment for some participants in the post-dosing phase, with some discussion of how different outcomes could have been if OCD had not interrupted or interfered with their psilocybin experience.

At the same time, the concept of ‘partial mystical experiences’ requires careful interpretation. It is possible that rather than simply being ‘less intense’ versions of full experiences, these may represent qualitatively different phenomenological states. The interference of OCD symptoms may not merely reduce mystical features but fundamentally alter the nature of the psilocybin experience. Additionally, neuroimaging studies have yielded mixed findings about alterations in 5-HT receptor availability in OCD (66), which may in turn account for the limited range and intensity of psilocybin-occasioned mystical experiences. Future research should examine whether: (1) higher doses might overcome OCD interference and/or possibly altered 5-HT receptor availability; (2) OCD-specific preparatory interventions could reduce interference; or (3) partial experiences may actually be more therapeutically relevant for OCD than full mystical experiences.

Overall, in terms of psilocybin’s acute impact on OCD, it was not common to see complete muting of symptoms. Rather, participants seem to waver between being preoccupied with arising obsessions and compulsive urges on one hand (see above), and feeling some cognitive distance from such symptoms on the other hand, most likely due to the increased ease of tapping into or engaging in acute adaptive (meta)cognitive processes such as cognitive defusion and mindfulness.

In particular, we noticed similarities between acute, adaptive (meta)cognitive processes during psilocybin and core processes of psychological flexibility discussed in ACT theory (48). Our participants described, to varying extents, moments during dosing in which they were able to reach a decentered, mentally expansive, or otherwise mindful state of awareness of their obsessions and compulsive urges as separate from their being, being able to detach from intrusive thoughts, urges, or other distressing internal experiences which they would previously be preoccupied with, and holding these in their expanded mindscape or sense of self. Some participants also reported being emboldened to approach difficult internal experiences (negative emotions or memories, obsessions) during dosing, sometimes with the support of their facilitators. These observations map onto desired approach behaviors often cultivated in ERP for OCD (67, 68).

Some of our participants’ reported acute insights and (meta)cognitive changes persisted post-dosing. For instance, some participants continue to redefine their relationship with OCD over the month after dosing, based on insights that their compulsions do not have a true functional, logical, or health-oriented purpose, or that appropriate risk-taking (e.g., approaching, rather than avoiding, OCD-related triggers) is paradoxically a viable path to liberating themselves from the constraints of OCD. These persisting shifts tended to have a self-efficacious, self-empowering quality, in contrast to the previous emotional heft of struggling against OCD in the pre-dosing phase.

While our analysis precludes causal inferences, some participants’ (meta)cognitive changes persisted alongside improvements in OCD symptoms within one month after dosing. More importantly, because some participants expressed clear attributions of post-dosing changes in (meta)cognitive processes and OCD symptoms to acute psilocybin effects, there is a plausible mechanistic hypothesis here; specifically, that it is possible that persisting (meta)cognitive changes, akin to those sought in ACT and ERP, contributed to OCD symptom change (and vice versa) in a synergistic manner. This bears out in the ACT and ERP literature on moderators and mediators of change in OCD treatment, in which symptom change and (meta)cognitive and behavioral change are shown to form a robust feedback loop feed in the cycle of symptom recovery (69–73).

We also found that other types of reported post-dosing changes tended to reinforce each other. For example, participants’ improved interpersonal functioning post-dosing appeared to boost positive emotionality and vice versa; both also appeared to be concurrent with, and plausibly an outcome of, improved OCD symptoms. In a sense, certain acute effects which persisted post-dosing may have catalyzed intertwined, downstream, longer-term benefits. We also noticed that some of these post-dosing changes were similar to what has been described in past psilocybin qualitative research with clinical samples, such as a deepened social connectedness, as well as the emboldened approach, rather than avoidance, of negative emotions (36–39, 42). In these instances, there is psilocybin-occasioned approach towards and connection with otherwise bothersome material, rather than avoiding or attempting to neutralize or eliminate such psychic content or symptoms.

Recent literature has theorized how principles of mindfulness and ACT can be integrated into transdiagnostic psilocybin treatment (74, 75). Our findings provide preliminary data supporting these possibilities, and highlight complementary processes shared by psilocybin and ACT (48) that may be worthwhile to examine in a comparative or augmentation trial. Additionally, our findings suggest a complementary role for ERP-relevant processes, capitalizing on an increased capacity for experiential approach to promote inhibitory learning processes (76–78). An integrated treatment approach (79) of, for example, psilocybin-assisted, ACT-informed ERP for OCD, may be a worthwhile focus of future trials. Because of the complexity of these components, more bottom-up, stakeholder-driven research is needed to build a pragmatic and integrative treatment protocol. Such research would likely emphasize the order of procedures that would maximize outcomes through a synthesis of stakeholder input and investigator theory on putative mechanisms, safety, efficacy, treatment adherence, feasibility, and durability of effects. Our research group is in the midst of conducting a mixed-methods, stakeholder-directed, treatment development study in pursuit of these aims, to arrive at a protocol that we shall evaluate in a future open-label, proof-of-concept trial. In this process, we are cognizant that the possibility of integrating psilocybin treatment with ACT and ERP for OCD emerged from an unstructured and non-directive approach to psychological support. This provides some impetus to include some element of non-directiveness in the integrated treatment, because such processes may not be as forthcoming with a wholly ACT- or ERP-based psychotherapy accompanying psilocybin dosing.

Several participants reported strengthened prior beliefs about the etiology of their OCD in the month following psilocybin dosing. This represents an interesting deviation from what may be predicted by the REBUS model, which hypothesizes that relaxed beliefs under the influence of psychedelics are likely to translate to long-term reconstitution, revision, or change in an individual’s belief systems and broader personality structure (80, 81). Notably, however, most of these prior attributions may already be considered adaptive in the context of OCD treatment, since they convey biopsychosocial, diathesis-stress perspectives about the causes of participants’ OCD that are amenable to subsequent change. It may be that psilocybin action loosens rigid, maladaptive, OCD-related beliefs and allowing adaptive, health-enhancing beliefs to emerge. However, it is unknown to what extent deepening or alterations of pre-existing beliefs about OCD (e.g., about how it was caused, developed, perpetuated, etc.) are related to acute psilocybin effects, as well as subacute variability in OCD symptom change (or lack of), among participants. Thus, psilocybin-occasioned changes in disorder-related causal attributions may need to be more rigorously assessed in future research, especially in light of emerging psychotherapy literature about the association between causal explanations and treatment response in OCD (82, 83).

Lastly, some participants had significant trauma history that emerged either in an obvious or metaphorical manner, occupying part of the psilocybin experience, and which prompted reliving as acute memories. These reports were spontaneous, given that we did not ask any trauma-related questions in our interview. While the potential of psilocybin for trauma reprocessing may be in a nascent stage of research at this time, these observations may warrant further investigation in a PTSD population, to stretch psilocybin’s purported role as a transdiagnostic treatment option for treatment-refractory conditions. Future research can also attempt to compare psilocybin treatment outcomes between individuals with OCD with and without trauma exposure, given emerging research on the role of trauma exposure impacting OCD symptom course and treatment response (84–87).

Limitations of this study include retrospective data collection at a single timepoint rather than a real-time micro-longitudinal approach, wherein the latter may allow for a more granular understanding of the evolution of immediate impacts of psilocybin over time. Our convenience sample of the first 12 completers may not represent the full range of psilocybin experiences in OCD, particularly those of participants who withdrew or declined to be interviewed. Additionally, given the integral aspects of diverse identities (as part of set) to the psilocybin experience, and the demographically homogenous sample in this study, future qualitative studies should strive to recruit narratives from a more diverse sample to adequately capture or demonstrate the dynamic interplay between person/set factors and psilocybin effects. Examples of such qualitative research include idiographic reports, albeit with entactogens (88, 89).

In closing, while not a focus of our current analysis, participants consistently expressed a desire to repeat the psilocybin experience, to recommend the trial to others with OCD, and having specific requests for higher and more doses. These remarks have informed the design of our current randomized, waitlist-controlled, single-blind trial of repeated psilocybin dosing coupled with unstructured and non-directive support for treatment-resistant OCD (46, 47), which we hope will be the next step to cultivate a rigorous program of innovative treatment outcome research for treatment-resistant OCD.

## Data Availability

The raw data supporting the conclusions of this article will be made available by the authors, without undue reservation.
