# Supplementary material for: Acute and post-dosing effects of single-dose psilocybin for obsessive-compulsive disorder in a randomized, double-blind, placebo-controlled trial: an interpretative phenomenological analysis
Source: Front Psychiatry. 2025 Dec 10;16:1726818. doi: 10.3389/fpsyt.2025.1726818 (PMC12728583; doi:10.3389/fpsyt.2025.1726818)
Supplement: Supplementary file 1 [file Table1.docx]

Supplementary Material

# Interview Guide

Introduction of interviewer

Hello, my name is _____________________________, and I have been asked to interview you today.

During the interview, I would like to discuss the following topics: the general nature of your experience, as well as subjects that may have arisen during the experience itself, such as life events, emotional experiences, insights, spiritual and/or religious experiences, and aspects of the experience concerning distress related to obsessive-compulsive disorder, or OCD.

If you want to take a break at any time to go to the bathroom or get a drink of water, just let me know.

| **Interview Guide** | **Clarification Questions** |
| --- | --- |
| **Pre-Dosing** | • Can you expand a little on  this?  • Can you tell me anything  else?  • Can you provide some  examples? |
| Can you give me a general sense of your life leading up to getting involved in the study. For example, can you tell me about your family life, your work life, your social life, etc.? |  |
| Generally, what is having OCD like for you?  (Optional follow-ups: What are your primary OCD-related concerns? What types of intrusive thoughts and obsessions do you struggle with? What compulsive behaviors do you engage in? How many hours a day does this bother you? ) |  |
| What impact has OCD had on your life?  (Optional follow-up: How has it affected your relationships, work life, aspirations, etc.?) |  |
| Can you describe your first memory of experiencing OCD symptoms? |  |
| What has been your path trying to address or treat the OCD? |  |
| How did you find out about the study?  Going into the study, what were your expectations? What were you hoping for or looking for? |  |
| **The Dosing Session** |  |
| Please describe in detail your experiences during the dosing session. |  |
| What perceptual changes, if any, did you experience (see, feel, hear)? |  |
| What internal changes, if any, did you experience? |  |
| What emotions arose during your experiences? |  |
| What memories arose during your experience? |  |
| During the session, what were your experiences of your body? |  |
| What insights or new understandings, if any, did you experience? |  |
| What, if any, impact did the OCD have on your experience of the session? |  |
| How would you describe your experiences after the session ended and later that evening? |  |
| **Post-Dosing** |  |
| For a moment, I ask you to think back to the time in your life before the treatment sessions began. I’m curious, in what ways do you feel being in the study has affected your life since undergoing the treatment sessions? |  |
| In what ways do you feel your participation in the study has affected your experience of OCD?  (Optional follow-ups: How has it affected the content of your specific obsessive concerns, intrusive thoughts, or compulsive behaviors, if at all? How has it changed the ways in which your OCD affects your relationships and other areas of your life, if at all?) |  |
| How has the study, if at all, given you insight into how OCD developed in your own life? |  |
| How has the study, if at all, affected your understanding of the nature of OCD or why it exists? |  |
| How has the study, if at all, affected your relationship to your thoughts or feelings? |  |
| **Other Questions** |  |
| Two study therapists were with you in the room during the treatment sessions. What was your experience of them and how did they affect your session? |  |
| Is there anything you wish had been different? |  |
| If given an opportunity, would you repeat the experience? |  |
| Is there anything else that you would like to share? |  |
| **Background and Wrap-Up Questions:**  What is your age?  What is your occupation?  Can you give me a brief outline of your family?  Do you have any questions for me? | |

# Full Version of Themes, Sub-Themes, Codes, Representative Participant Quotes, and Interpretations

| **Theme/Sub-Theme** | **Code** | **Representative Participant Quote** | **Interpretation** |
| --- | --- | --- | --- |
| **Influences on Psilocybin Experience**  *Set* | Symptom Relief (*n* = 11) | P17: “I was really, I guess, hopeful… I think I was really hopeful, like, ‘Oh, this will cure it, this will fix it.’” | Intentions centered on relief vs. cure; for latter, expectation calibration during preparation shaped subsequent engagement. |
| **Influences on Psilocybin Experience**  *Set* | Explore and Learn (*n* = 8) | P5: “I wanted to have an experience where the OCD turned off… I was very willing to stare into the eyes of the monster and I wanted to see what was really in there. I wanted to know if I cut the noise out, what's left. And if I was able to dial in to… the truth of me, what's left and… what's gone... That was my hope, and even, expectation.” | Openness to discovery primed metacognitive stance and curiosity-driven approach behaviors. |
| **Influences on Psilocybin Experience**  *Setting* | Comfortable Dosing Room (*n* = 9) | P10: “Another thing for me was that I don't think that I was prepared mentally to be in the research unit, because I had never actually stayed or been in a psych ward before. But on the flip side, that helped tremendously… having gone through that first week. Coming back a second time, I knew what to expect… So, I had less discomfort, anxiety around being in the unit the second time.” | Familiar, well-tuned space – especially during open-label dosing – reduced anticipatory anxiety and supported immersion into dosing experience. |
| **Influences on Psilocybin Experience**  *Setting* | Facilitator Effects (*n* = 10) | P13: “I was holding [my male facilitator’s] hand… He felt like my parent. [At the same time,] he actually said… I didn't even say anything… He said that he felt very paternal in that moment. It was very interesting. So, I was holding onto his hand really tightly and he kind of just, like, lifted up the headphones, and was like, ‘Alright, we're going to let you swim now,’ and relaxed his grip and let me go. And it was like, ‘Boom!’ I was underwater. It was crazy… It was like I'm in the warmest, coziest ocean imaginable. And I felt like I was about 15 or 20 feet below the surface and I could hear the water and the distance above and just kind of in the background… It was just so key. It felt very peaceful, and it didn't last very long, but I was in this, like, warm ocean.” | Attuned, non-directive support functioned as a stabilizing scaffold enabling deeper surrender to or re-engagement with dosing experience. |
| **Influences on Psilocybin Experience**  *Setting* | Music Effects (*n* = 11) | P16: “The music felt very intense. It was one of the better musical experiences of my life, I would say. If I had any sort of nerves about going into it… I would say those dispersed fairly quickly, because it was just, like, joyful and pleasant.”  P17: “I just don't think my mind was, like, able to register it. Like, when I was listening to instrumentals that were just, like, piano [music], I was like, ‘Okay. That's music.’ But then when I started hearing, like, singing from voices, I was, like, ‘That doesn't make sense.’ I don't know. It was just different. Like, I didn't wanna hear singing voices.” | Musical arc entrained affect and imagery; vocals sometimes overloaded cognition, highlighting set sensitivity. |
| **Acute Effects**  *Acute Perceptual Effects* | Acute Physiological / Somatic Effects (*n* = 9) | P10: “At one point – going back to [what I was describing as an] unburdening – visually, I could see like this light, this energy, this weight lifting. And it was kind of this pulse, this white energy from my chest, pretty much from the top of my body. I mean, my eyes were closed, but I had a sense of my physical body lightening, it was like a literal representation of weight lifting.”  P16: “I remember the day [my father] died, I just was, like, lying on the floor and just felt this extreme sense of sadness, this deep weight. And I had not felt that since, and that was, you know, six years ago or something like that. And when I was in the psilocybin treatment, I was, like, just crying, like this full-body sadness. I hadn’t felt that in probably six years.” | Somatic shifts (relaxation, lightness, or discomfort) co-occurred with meaning-making and affective release. |
| **Acute Effects**  *Acute Perceptual Effects* | Acute Visual Effects (*n* = 11) | P17: “A significant part of my psilocybin experience was seeing this entity… Whenever they would show up during my experience, they very much were showing me that life is full of paths that I can take… [Because] I guess OCD can make you feel like… you don't have that much power or control over your choices and stuff. So that entity was kind of just showing me that I still have the power to make decisions and choices.”  P15: “From ages 7 to 17, my father was a pretty bad alcoholic… And one of the things he did with me… was to take me out to teach me how to drive when he was drunk and when I wasn't old enough to drive. And I had a vision of that experience… I had a sense that he was trying to do that over again, and he was trying to do it right. He wasn't drunk. And then I just had this overwhelming sense of love from him, and that he was sorry for, you know, everything that had happened.”  P3: “I was lying on a bed, almost felt like a hospital bed, or a death bed. And it was a white room. My parents were next to me, on the bed… I was able to feel love from them. This was, I think, the most powerful part of the trip. I was able to feel like they were compassionate toward me, as if I were dying and they were right there supporting me… My relationship with them has been strained throughout the last 10 years [because] of [their] divorce and my OCD. So, I haven't really felt love ever toward them, but the key thing I learned from this is that everything that they do for me is out of love. All the crap that we have with our relationship, I learned that that's not the whole relationship. That's just part of it. It's just a layer of it. And deep down, there's love.”  P6: “I remember distinctly saying, ‘This is not for the faint of heart.’ It was like I'd been pulled into the sea in this crazy storm, and somehow, I came out of it and now I knew how to swim. And at that point, I could sort of control the experience… And, uh, it was, like, ‘Okay, it's time. It's time to do work again. Put the headphones and eyeshades back on, lay back, and just dive deep into myself and do more of that.’”  P3: “There's a little OCD thing that happened. I don't know if I tasted it in reality, but, um, I had a sensation of tasting the essential oils [by the side of the dosing bed]. But I started freaking out, like, ‘Oh, did I drink it?’ And I saw a visual of, like, poison entering my body, through my mouth. And I noticed I started going, ‘Oh, did I just ruin the whole trip?’ Um, but it was a poison visual, like, in my mouth, entering my body. It was a little scary.” | Rich imagery catalyzed insights and positive emotions; occasionally amplified fear or activated obsessive content. |
| **Acute Effects**  *Acute (Meta)Cognitive Effects* | Acute Insights (*n* = 10) | P16: “I was walking on the beach, and suddenly, I knew that the beach was, like, in my brain, and I picked up a shell and looked at the shell, and the shell was pulsing and sending off all of these pulsing sounds. And I realized that the shell itself was my OCD… And I said, ‘Oh, here's my OCD.’ Um, I think that the idea [was] that OCD might not be this malicious force and is just something as normal as, you know, an ecological force. I find it comforting, I guess. That felt like a nice thing for me.”  P12: “What is probably one of the most significant [dosing insights] was this idea that I can still lead a full emotional life. We're still thinking about people in my life, even if there are intrusive thoughts.”  P13: “There's definitely this glimpse, at least peripherally of… maybe some greater truth. I wasn't like, ‘Oh my God, I've seen the light.’ But there's definitely this sense of, ‘Okay, there's a deeper something that's connecting all of us.’” | Spontaneous insights emerged, representing a more compassionate or mindful stance on OCD, or other personal domains or life events. |
| **Acute Effects**  *Acute (Meta)Cognitive Effects* | Other Acute Adaptive (Meta) Cognitive Effects (*n* = 11) | P5: “There was this mental war going on in my mind… ‘How do I approach the psilocybin experience?’ This is…how my mind works normally, so it really makes sense that this is part of the experience. So, I put on the eyeshades, right? And I lay back down, and I'm trying to open myself up to go deeper… Part of me was thinking, ‘You need to purposefully, actively go deeper.’ And the other part of me was, ‘No, you go deeper by just relaxing and letting it pass, like floating downstream.’ And these two parts of my mind were going back and forth, and it created this battle scene in my mind of, ‘What is the approach?’ … [It’s] just exactly how my OCD talks. Um, obviously, there is a way to go deeper, and obviously, it's the side where, um, you allow yourself to relax into it. That's how you sink into it... But there was this other part of me, um, that was, um, desperately trying to be relevant… that was trying to control it and be a part of it… And so, it was…this really strong grip, [but] it wasn't as strong as it normally is.”  P12: “At a certain moment in the experience… I went over to the picture of my sister, and I was looking at it very carefully… It was pleasant, and I was appreciating the picture aesthetically… It was interesting, because I did have intrusive thoughts, but nonetheless, I really felt, ‘There’s my sister.” She had such a broad smile in the picture, she's so happy. I felt her personality. I thought about how much I love her. I thought about what a cool person she is… Yeah. So, I had a very positive emotional experience, and that involves thinking about her. So, what that said to me was that, ‘I can have this – these thoughts that I want so much to have – even if there are intrusive thoughts.’ There's not an either/or equation.” | Decentering/defusion enabled flexible attention away from compulsive urges and cognitive rigidity. |
| **Acute Effects**  *Acute (Meta)Cognitive Effects* | Acute Experiential Approach (*n* = 9) | P17: “Yeah, I was trying to [control the onset of psilocybin], which was very much overwhelming me, which I've learned now [that] my brain just likes to have complete control and understanding of things. So, when I wasn't able to, like, have a complete control and understanding of things, my brain was, like, almost, like, short-circuiting. Like, my brain was, like, ‘I cannot understand this. Why am I not able to understand this?’ And when I was able to just let it go and not think about it anymore, I very much went from a very intense position to being very, very calm.”  P3: “All that…challenging [of] beliefs that I was doing… I somehow pulled…from deep within strength and courage to take a risk. Even though I wasn't able to, like, fully let go, I still took a little risk. And that taught me that I have that strength inside, and I can do that.”  P8: “I felt partially responsible for that because I had actually…reported to the school that there was somebody bringing drugs into campus…that students would [overdose] on… And over the years, I've just kind of blurred the reality where I thought, like, ‘Man, am I responsible for him getting kicked out? Am I responsible for him committing suicide?’ And the answer is, ‘No, I never mentioned him…’ Nobody was ratted on. We were trying to keep people safe. Um, but [that] haunted me my whole life and the truth got away from me, you know? I didn't know what was true. I [used to think], like… ‘Maybe I effectively killed this kid.’” | ‘Going with’ difficult experiential content paradoxically decreased arousal, consistent with approach-oriented extinction learning in CBT/ERP for OCD. |
| **Acute Effects**  *Acute (Meta)Cognitive Effects* | Acute Memories (*n* = 7) | P12: “I had a surprising image of entering…a series of vaginas, as if they were [unfurling] petals of a flower. It wasn't sexual… It didn't feel like a birthing image… It was really wholesome. It evoked a lot of, like, very strong appreciation of motherhood. It made me very appreciative of all the women in my life. It was preceded by an image of somebody who accompanied me to the doctor's office, which actually occurred... And she herself is a mother, and has complicated relations with her own mother. I don't know, it was just healing. It was, like, just a very strong appreciation of motherhood, and as I said, the women in my life in general.” | Revisited autobiographical scenes supported corrective emotional experiences and narrative integration. |
| **Acute Effects**  *Acute Emotional Effects* | Acute Positive Emotions (*n* = 8) | P10: “I remember the moment because it was a particular track within the playlist… There was this indigenous elder singing… I put the headphones back on and…I could visually see his head next to mine. And as he was singing… I saw blue and white [colors] just kind of stream across my mind…or across my [inner visual] field… It was just a really blissful moment.” | Love, awe, safety, gratitude broadened repertoire and reinforced adaptive appraisals. |
| **Acute Effects**  *Acute Emotional Effects* | Acute Negative Emotions (*n* = 8) | P17: “If there was, like, a sadness about anything, it was a sadness about realizing that I'm very disconnected from the people that I love, and things that I love to do in life. So, it was very sad how much I had been missing out in life.” | Distress and grief surfaced and were tolerable; coexisted with positive states without avoidance. |
| **Acute Effects**  *Acute Impact of OCD* | Acute Interference by OCD (*n* = 8) | P17: “The big moments that I think of where I was avoiding was when I would take off my headphones and the [eyeshades], because I didn't wanna see that [entity] anymore. And so, I mean, I guess that moment could be a very big example of my typical avoidance.” | Obsessions/compulsions intruded, fragmenting immersion; illustrates partial mystical profiles in OCD. |
| **Acute Effects**  *Acute Impact of OCD* | Acute Muting of OCD (*n* = 8) | P15: “I don't think [OCD really entered my psilocybin experience]. I had…a very real sense that there was some kind of surgery going on in my brain… I just had this very real sense that something was being…taken out of my brain. And I was hoping it was the OCD.” | Transient ‘turning off’ or reduced salience of OCD, which created window for fuller experiencing of other aspects of dosing experience. |
| **Post-Dosing Changes in OCD**  *Post-Dosing Changes in Symptoms* | Changes in Obsessions (*n* = 8) | P10: “The OCD is still present… Coming out of [the psilocybin] session, the day after, initially, I felt as if all of the other [OCD] themes were minimized, even if the main hyperawareness one was still present… Eventually settling back into my daily life and routine, these other thoughts still…kind of come back, but it's [a] normal kind of feeling and thinking.”  P8: “Every day, since [the] day [of dosing], has been better than the one before it, because every day since that day has been just more freedom [from my OCD], just more peace.”  P8: “The experience of having died and the experience of being a tree were both equally important in different ways. If I hadn't had both, I don't know if [the effects on my OCD] would have, um, stuck so well… Both of those were really, really important takeaways.” | Content often persisted, but stance softened; some achieved remission, suggesting heterogeneous trajectories. |
| **Post-Dosing Changes in OCD**  *Post-Dosing Changes in Symptoms* | Changes in Compulsions (*n* = 8) | P9: “It is much easier now for me to say, ‘I'm just going to push past this.’ I was taking something out of the trunk of my car, and my hand touched something in my trunk that before, I would have been compelled to go into the store and wash my hands. And not only did I not do that, I was able to touch other things in my car with my hand. And it was fine. I didn't think twice about it, and I actually made an effort to touch other things so that… I wouldn't have to think about it anymore. I mean, it was just different from the past.” | Reduced rituals via increased tolerance and agency; occasional full remission reported. |
| **Post-Dosing Changes in OCD**  *Post-Dosing Changes in Perceptions of OCD* | Strengthened Prior Beliefs about OCD (*n* = 8) | P16: “I think there is something that is structural, probably, happening. What I suspect is there's a lot of things that can contribute to OCD. Genetics is probably one of them. I'm also like a child who would qualify for, like, PANDAS, and I was born with strep. They thought I was going to die. I was born with strep and spinal meningitis... I have, like, very, very, very clear genetics, and also, like, some of the early [perinatal events].” | Psilocybin consolidated adaptive biopsychosocial attributions rather than globally relaxing beliefs. |
| **Post-Dosing Changes in OCD**  *Post-Dosing Changes in Perceptions of OCD* | Destigmatizing OCD (*n* = 7) | P3: “[The psilocybin] didn't fix the OCD… But it taught me, ‘[The OCD is] just a part of you.’ Like, you can learn to live with it and almost learn to love it. And now I'm almost proud of it, and not really ashamed of it… The stigma and shame [are] going away… Now, it's a possibility for me to talk about it with someone… It's just a part of me… I realized I have all these redeeming qualities within myself, there's a lot of good stuff about me, and the OCD, it can be good too. It's just something that I have to learn to work with, and I can still work on it and make it better.” | Shame decreased, acceptance and disclosure increased; identity integration over concealment. |
| **Post-Dosing Changes Beyond OCD Symptoms**  *Post-Dosing (Meta)Cognitive Changes* | Post-Dosing Insights (*n* = 9) | P3: “Sometimes moving forward can be risky, but less risky than going backwards… I have to work myself out of my comfort zone by…pushing the boundaries slowly, do things that make me uncomfortable…and then just keep working on that and I'll grow and I'll feel better and I'll improve. [It has to do with] accepting that the OCD is [still] here. I have an interest now in doing ACT therapy. I felt a lot of the stuff gained was, ‘Alright, I can go on with my life with this stuff…’ I just have to learn to…do stuff to improve, but also…live with [OCD] and accept that…[even though] it might be part of me forever… I'll still be able to go on…and have a fulfilling life.”  P17: “Avoidance was a big thing that I learned that I do… Sometimes, I just have to be willing to lean into discomfort and lean into the unknown. And life is just a big unknown, so you just have to go with it and roll with it…[and] good things can come of it. I'll never know what I was gonna see with that [entity], like where it wanted to take me, [because] I didn't go with it. But at the same time…it showed me that progress can be made outside of the psychedelic… There's also that realization that no one thing is gonna fix you, and that maybe it's just a constant work-in-progress.”  P8: “I do think there's something chemically that does work… There is something that turns off OCD with psilocybin.” | Actionable realizations about living with OCD (enacting values, taking risks) anchored ongoing change. |
| **Post-Dosing Changes Beyond OCD Symptoms**  *Post-Dosing (Meta)Cognitive Changes* | Other Post-Dosing Adaptive (Meta) Cognitive Effects (*n* = 9) | P9: “I think [the study] has affected my life positively, and I have more optimism than I did before, that I can make…changes. [Life] would be better for me… My life doesn't have to be so dominated by [my OCD].”  P13: “[There’s] kind of this deeper sense that life is worth it and…more of a shift towards looking towards my future as opposed to dwelling on the past.”  P18: “I wrote [in my journal], ‘Sensation of feeling in alignment, because I'm in control, not OCD. The OCD's still there, but I have the choice to ignore it. I have the choice to act in accordance with my values and my best interests, like my health, my happiness, my will of being.’”  P3: “I've learned that there's…two sides to everything… So, yes, it was fearful and anxiety-provoking…a lot of the session, but I'm able to use a lot of that to help me… [The psilocybin session] showed me…what I can do. It allowed me to face [my OCD], and that was the biggest exposure that I've done.”  P6: “There was…this…distinct feeling [during dosing] that the feelings are…real, and they're there, but they're…also objects, and quite feel that way still now. I view my thoughts and emotions as more that way [now], as things that appear in consciousness, and I don't necessarily have total control over all of them. I do think that has helped, if nothing else… [using] that framework…as a starting point.” | Greater psychological flexibility, self-efficacy, and resilience generalized beyond OCD triggers. |
| **Post-Dosing Changes Beyond OCD Symptoms**  *Post-Dosing (Meta)Cognitive Changes* | Post-Dosing Experiential Approach (*n* = 8) | P18: “I had the insight…‘[My perfectionism] is getting in the way of having a…train of thought.’ It makes sense that it'd be difficult…to write a paper if I keep interrupting myself [by rereading and rewriting]. So, I've been kind of training that mental muscle of just writing free-flow, not judging it, just letting it flow out.” | Daily-life ERP-like behaviors (approach, non-avoidance) independently enacted. |
| **Post-Dosing Changes Beyond OCD Symptoms**  *Other Post-Dosing Changes* | Interpersonal Functioning Post-Dosing (*n* = 7) | P6: “If it wasn't clear before, it is super clear now… Being there for her is the…top priority… Six months ago, if you'd asked me if I ever wanted to be like vice-president of my company, I would have said, ‘Yeah, maybe someday…’ And if you ask now, the answer is, ‘No, [because of] the trade-off…’ It's what I would have to give up that is not worth that trade.”  P8: “We're having some tough medical stuff with our son… My wife, yesterday, spent a lot of the evening…crying…about it. And I could actually be there for her instead of her…worry[ing] about how I'm doing… She could actually take time and space to have emotions of her own…and I could be there and support her and help her through that… Finally, I can give back and help her, you know? It felt incredible. [It] never, ever, ever would have happened before this study.” | Improved connectedness and caregiving capacity emerged alongside symptom relief. |
